# Editorial: Highlights for Cardiovascular Therapeutics in 2021 – Trained Immunity, Immunometabolism, Gender Differences of Cardiovascular Diseases, and Novel Targets of Cardiovascular Therapeutics

**DOI:** 10.3389/fcvm.2022.892288

**Published:** 2022-04-27

**Authors:** William Y. Yang, Bonnie Nguyen, Sheng Wu, Jun Yu, Hong Wang, Xiaofeng Yang

**Affiliations:** ^1^Centers for Metabolic Disease Research, Department of Cardiovascular Sciences, Temple University Lewis Katz School of Medicine, Philadelphia, PA, United States; ^2^Cardiovascular Research, Department of Cardiovascular Sciences, Temple University Lewis Katz School of Medicine, Philadelphia, PA, United States

**Keywords:** trained immunity, cardiovascular therapeutics, cardiovascular diseases, gender differences, novel targets

## Introduction

Due to the highly collaborative efforts of the authors, editorial office, and editorial team, the Frontiers in Cardiovascular Medicine - Cardiovascular Therapeutics section has made significant achievements in 2021, with a five-fold increase in manuscript submission. We all greatly appreciate it. In 2022, we will continue our efforts to build an outstanding platform for cardiologists and translational cardiovascular scientists to exchange novel findings and data in clinical cardiology and cardiovascular therapeutic fields. Here, we would like to highlight some excellent articles published in our section as well as new progress in the field with significant potential in cardiovascular therapeutics. In addition, these highlights may also serve as the foundation for some new special topics in our Cardiovascular Therapeutics section in 2022.

## Trained Immunity, an Innate Immune Memory, is a New Inflammation Amplifying Mechanism

Cardiovascular disease (CVD) is a leading cause of death in the USA and worldwide. Numerous risk factors for triggering the onset and promoting CVD progression have been identified, including hyperlipidemia (1), hyperglycemia, hyperhomocysteinemia (2), smoking, metabolic syndrome, hypertension, obesity (3–5), and infections (1, 6–9). On the other hand, many danger-associated molecular patterns (DAMPs) and conditional DAMPs (10) have been characterized in triggering the recruitment of monocytes, macrophages, T cells, B cells, and other immune cells into arteries to amplify the atherosclerotic process, suggesting that innate and adaptive immunity contribute to the atherosclerotic plaque formation. However, several important issues remain poorly characterized: *first*, what compounds in the environment and endogenous metabolic process in the host are qualified to become risk factors to stimulate atherosclerotic CVD (11); *second*, many disease risk factors are synergistic in promoting atherosclerotic CVD, including *a)* hyperhomocysteinemia and hyperlipidemia (12, 13); *b)* hyperhomocysteinemia and hyperglycemia (14, 15); *c)* multiple DAMPs stimulation in chronic kidney disease (CKD) (16–19); *d)* hyperlipidemia and CKD (17); *e)* COVID-19 infection (coronavirus disease-2019, a viral respiratory illness caused by the severe acute respiratory syndrome-coronavirus-2, SARS-CoV-2) (20) and CVD; and *f)* sex hormone dysfunction and CVD (21); *third*, what cellular processes contribute to the quality and synergy of disease risk factors? Innate immune cells can develop exacerbated long-term immune responses and inflammatory phenotype following brief exposure to endogenous or exogenous DAMPs, which results in a primed and significantly enhanced inflammatory response toward a second challenge after returning to a non-activated state. This phenomenon is known as innate immune memory or trained immunity (TI). TI is important for host defense, vaccine response, and promoting the pathogenesis of chronic inflammations including metabolic CVDs such as atherosclerosis. In contrast to the memory function in the adaptive immune system with special cell subsets to carry out memory function such as memory T cells and memory B cells (22), TI can occur in innate immune cells such as monocytes/macrophages (23), natural killer cells, endothelial cells (ECs) (6, 11, 24), vascular smooth muscle cells (25, 26), and nonimmune cells, such as fibroblasts (7) and hepatocytes (27, 28). Of note, we recently reported that CD4^+^Foxp3^+^ regulatory T cells (Tregs) have many active innate immunity pathways (29, 30), and can sustain their immunosuppressive functions (31) in a proinflammatory atherogenic environment, although Tregs plasticity in atherosclerosis has been reported (32, 33). It has been reported that increased energy metabolism pathways and electron transport chain (34), including glycolysis, acetyl-CoA generation, mevalonate synthesis, glutaminolysis, and epigenetic modification (11, 35), contribute significantly to the establishment of TI. Extensive characterization of TI relative to CVD would provide novel insights into CVD pathogenesis and new therapeutic targets. To demonstrate the therapeutic potential of inhibiting new TI-related metabolic pathways such as glycolysis, Gager et al. reported that sodium–glucose co-transporter 2 (SGLT2) inhibitors, an emerging class of glucose-lowering drugs, have become increasingly relevant for the treatment and prevention of heart failure (HF). SGLT2 inhibitors are associated with improved cardiovascular outcomes in patients with HF (Gager et al.). In addition, Chen et al. reported that 2-Deoxy-D-glucose (2-DG), a glycolysis inhibitor, can alleviate cardiac fibrosis after myocardial infarction (MI) (36).

## Trained Immunity May Be Underlying the Synergy of Multiple Risk Factors in Promoting the Pathogenesis of Cardiovascular Disease

A significant attribute of trained immunity in promoting cardiovascular diseases is that TI pathways' synergy among multiple risk factors contributes to CVD progression, which provides novel guidance for cardiovascular therapeutics. A recent report showed that COVID-19 may predispose patients to thrombotic disease, both in the venous and arterial circulations, attributed to excessive inflammation, platelet activation, endothelial dysfunction, and stasis (37). These findings emphasize that inflammation induced by COVID-19 infection may serve as the first stimuli in the TI setting and predispose patients to thrombotic disease (second stimuli). Zhao et al. reported that antithrombotic management for atrial fibrillation (AF) in patients undergoing percutaneous coronary intervention or with acute coronary syndrome [an evidence-based update combined antithrombotic regimens for AF in coronary artery disease patients, particularly in acute coronary syndrome (ACS) patients or patients undergoing percutaneous coronary intervention (PCI)], presents a great challenge in the real-world clinical scenario. The results of these studies have impacted the recommendations of current international guidelines, which favor a dual antithrombotic therapy (DAT) with a non-vitamin K antagonist (VKA) oral anticoagulant (NOAC) and classic antiplatelet drug and potential inflammasome inhibitor (38) P2Y12 inhibitor (39) (especially clopidogrel) in the clinical setting. Aspirin, a nonsteroidal anti-inflammatory drug, can be administered during the periprocedural period, while triple antithrombotic therapy (TAT) treatment duration should be as short as possible (Zhao et al.). Similarly, Bitar et al. conducted a systematic review of 326 articles and a meta-analysis on eight randomized clinical trials to evaluate the efficacy and safety of direct oral anticoagulants (DOACs) vs. warfarin (brand names Coumadin and Jantoven) in the treatment of AF and valvular heart disease (VHD). They found that DOACs remained with similar efficacy and safety compared to warfarin in thromboprophylaxis for AF and VHD (Bitar et al.). Since the interplay has been reported between inflammation and thrombosis in cardiovascular pathology (40), once again, these findings support our argument that blocking trained immunity as an inflammation amplifying pathway could be a benefit for cardiovascular therapies.

Insulin resistance (IR) has been identified as a risk factor and metabolic stress to accelerate vascular dysfunction and cardiovascular disease relative to type 2 diabetes mellitus (T2DM) (41). Controversies concerning the association between insulin therapy and atherosclerotic lesions in T2DM remain. Ke et al. investigated whether insulin therapy in T2DM patients is linked with the increased risk of carotid atherosclerosis, by retrospectively enrolling 2,356 hospitalized patients with T2DM, including 1,716 subjects receiving insulin therapy and 640 subjects without insulin therapy. This report showed that insulin therapy is associated with a markedly increased risk of carotid atherosclerotic lesions in T2DM, partly contributing to the more severe insulin resistance in T2DM patients receiving insulin therapy (Ke et al.). These findings correlate well with the previous findings from other teams that hyperinsulinemia is strongly associated with T2DM and is an early indicator of metabolic dysfunction (42). A potential mechanism underlying these findings may be insulin signaling and IR functions in promoting trained immunity in macrophages through metabolic and epigenetic changes (43).

## NAD+ As an Immunomodulator May Inhibit Trained Immunity

Nicotinamide adenine dinucleotide (NAD+) has a direct inhibitory effect on poly(ADP-ribose) polymerase 1 (PARP-1) (44) and can prevent pro-inflammatory cytokines' over-activation. Increasing the NAD+ level will also stabilize telomeres, which positively impacts immune cells' function (45). We recently reported that liver ischemia reperfusion injury (IRI) is enhanced by trained immunity but is attenuated in the deficiency of pro-inflammatory DAMP/conditional DAMP (10) sensor (38) caspase-1/caspase-11 (human caspase-4) pathways in gene knockout mice (27, 46). Xiao et al. reported the cardioprotective properties of known agents in rat IRI model under clinically relevant conditions: only the NAD precursor nicotinamide riboside (NR) reduces myocardial infarct size in the presence of fentanyl (a synthetic opioid that is 80–100 times stronger than morphine), midazolam (produces sleepiness or drowsiness and relieves anxiety), and cangrelor (purinergic receptor P2Y_12_ inhibitor), but not propofol (P2Y_12_ antagonist). This observational study suggests that NR is a promising cardioprotective agent to target cardiac IRI in clinical conditions employing opioid agonists, benzodiazepines, and platelet P2Y_12_ inhibitors, but not propofol (Xiao et al.).

## Rna Therapeutics Becomes a New Front in Cardiovascular Therapeutics and Regulators for Trained Immunity

The unprecedented expansion of data and information on RNA biology has led to new RNA classes with unique functions and unexpected modifications. The biggest challenge is to transfer the large number of findings in basic RNA biology into corresponding clinical RNA-based therapeutics. Lately, this research has begun to yield positive outcomes. Schellinger et al. reviewed significant progress on this front. RNA drugs advance to the final phases of clinical trials or even receive U.S. Food and Drug Administration (FDA) approval. Furthermore, the introduction of the RNA-guided gene-editing technology, the clustered regularly interspaced short palindromic repeats (CRISPR), and the advances in the delivery of messenger RNAs have triggered a significant progression in the field of RNA-therapeutics. Short interfering RNAs and antisense oligonucleotides especially are promising examples for novel categories of therapeutics. However, several issues need to be resolved, including intracellular delivery, toxicity, and immune responses, before utilizing RNAs in a clinical setting. Schellinger et al. provided an overview of opportunities and challenges for clinical translation of RNA-based therapeutics, emphasizing advances in novel delivery technologies and abdominal aortic aneurysm (AAA) disease where non-coding RNAs have been shown to play a crucial regulatory role (Schellinger et al.). To facilitate the studies on TI, a comprehensive database (47) was established, including 118 trained immunity regulators. Further characterization of master regulators, such as NADPH oxidase 2 (NOX2) (48), nuclear factor erythroid 2 (EFE2) like basic leucine zipper (bZIP) transcription factor 2 (NRF2) (20), hypoxia inducible factor 1 subunit alpha (HIF1a), mechanistic target of rapamycin kinase (mTOR), and SET domain containing 7 histone lysine methyltransferase (SET7) (49), will allow us to use RNA therapeutics to inhibit TI facilitated cardiovascular diseases.

## Cell Death May Orchestrate a Balance Between Trained Immunity- Associated Chronic Cardiovascular Inflammation and Resolution

Cell death may orchestrate a balance between trained immunity-promoted chronic cardiovascular inflammation and resolution (50). Several new forms of cell death have been identified recently in infections, inflammation (51), cancers, and cardiovascular diseases (52), including panoptosis (53), pyroptosis (38), necroptosis, and ferroptosis (54). Ferroptosis is a type of regulated necrosis triggered by iron toxicity, lipid peroxidation, and plasma membrane damage. It is distinct from apoptosis, necroptosis, autophagy, and other types of cell death in morphology and function. The upstream inducers of ferroptosis can be divided into two categories (biological and chemical) and activate two major pathways (the extrinsic/transporter and the intrinsic/enzymatic pathways). Excessive or deficient ferroptotic cell death is implicated in a growing list of physiological and pathophysiological processes, coupled to a dysregulated immune response (55). Ferroptosis is regulated by various factors and controlled by several mechanisms, including mitochondrial activity and metabolism of iron, lipid, and amino acids. Accumulating evidence shows that ferroptosis is closely related to a majority of CVDs, including cardiomyopathy, myocardial infarction, ischemia/reperfusion injury, heart failure, and atherosclerosis. Hu et al. summarized the current status of ferroptosis and discusses ferroptosis as a potential therapeutic target for CVDs (Hu et al.). Further characterization of novel cell death pathways would lead to the future development of therapeutics for CVD.

## Sex Hormones May Modulate Trained Immunity and Cardiovascular Diseases

Most studies on CVD have been conducted on male subjects, and assumed that women and men have similar physiological responses. However, the effects of sex hormones and their respective receptors in modulating cardiovascular functions are not uniform between men and women. The women (56) or female rodents (57) were associated with elevated androgen experience insulin resistance and increased risk of CVD (58). In contrast, men are at higher risk for CVD than women before the ages of 60 years old (59). It has been reported that low estrogen levels in younger females are associated with an increased risk of CVD. In addition, decreased estrogen levels after menopause are associated with dyslipidemia, increased blood pressure, and CVD. Moreover, pregnancy complications, including gestational diabetes and preeclampsia, and endocrine disorders such as polycystic ovarian syndrome promote CVD (60, 61). A recent report showed distinct sex differences in neutrophil biology related to responses to type I interferons (IFNs), immunometabolism, and maturation status that may have prominent functional and pathogenic implications (62). More directly, 17β-Estradiol was reported to promote TI in females against sepsis via regulating nucleus translocation of pro-inflammatory transcription factor RelB (48). Testosterone was identified to play a very important role in modulating the innate and adaptive immune systems (63). Further characterization of the functions of sex hormones in modulating TI relative to CVD pathogenesis would significantly improve our understanding of sex differences in CVDs and lead to the future development of therapeutics for CVD.

## Author Contributions

WY, BN, SW, JY, and HW provided material input and participated in writing. XY supervised and edited the manuscript. All authors contributed to the article and approved the submitted version.

## Conflict of Interest

The authors declare that the research was conducted in the absence of any commercial or financial relationships that could be construed as a potential conflict of interest.

## Publisher's Note

All claims expressed in this article are solely those of the authors and do not necessarily represent those of their affiliated organizations, or those of the publisher, the editors and the reviewers. Any product that may be evaluated in this article, or claim that may be made by its manufacturer, is not guaranteed or endorsed by the publisher.
